# Guy's Hospital

**Published:** 1832-01

**Authors:** 


### GUY'S HOSPITAL.

### *Case of Scrotal Hernia, treated by* Mr. Bransby Cooper.*

John May, ætat. forty-eight, a carpenter, came into Guy's Hospital on the morning of the 26th of October, under the care of B. Cooper, Esq., with a scrotal hernia of the right side: he had been subject to hernia for the last twenty-five years, and wore a truss occasionally during that time. He has been in the habit of drinking three or four bottles of port wine daily at the Isle of France, and since his arrival in England (a fortnight ago,) he has drank three or four gallons of porter a day. The hernia often came down, and was easily returned. On the evening of the 25th, whilst intoxicated, (in which state he had been for the last five days,) the hernia descended more than usual, and remained so the whole night, with the hard truss pressing on its neck. On the morning of the descent of the hernia, his bowels were freely opened. Early on the morning of the 26th, he went to a surgeon, who tried to return it, but was unable to do so: he then bled him to ℥xxxij., when the patient fainted: upon his recovery, the surgeon advised him to apply to Guy's Hospital, and, as Mr. Cooper was there, he saw him immediately on his admission.

He described himself to have suffered from nausea, vomiting, and hiccup, early in the morning; but these symptoms had now subsided.

When Mr. Cooper saw him, he had pain in the bowels and tenesmus. The taxis was employed without any effect: he was then placed in a warm bath, in which he remained for a quarter of an hour, during which time the taxis was again employed, but in vain: from thence he was removed to bed, and a purgative enema administered, which quickly returned without bringing away any fæces. The ice mixture was then applied for half an hour. At about one o'clock, the hiccup returned; the abdomen became tender to the touch; the tumor increased in size, and was very painful; the face was flushed, with pain in the head and eyes; the tongue moist, and its papillæ raised; the pulse full, and very frequent.

Mr. Cooper now consulted with his colleagues, Mr. Morgan and Mr. Callaway, as to the propriety of operating, and having determined in the affirmative, a proposal to that effect was made

* Dr. James Johnson was kind enough to transmit this report to us.—Editor.

by Mr. Cooper to the patient, to which he readily consented. At two o'clock, the patient was taken into the operating theatre, and, being placed on a convenient table, Mr. Cooper commenced the operation, by making an incision from opposite to the external ring, about two thirds of the length of the tumor; not, as usual, continuing the incision the whole length, in consequence of the great size of the hernia, a similar incision was then made through the fascia spermatica externa and cremaster muscle; the fascia spermatica interna, which, in this instance, was particularly strong, was then divided: at its lower part a quantity of fluid escaped, which led the operator, at the moment, to believe that the hernial sac was opened; but it proved to be a hydrocele projecting the tunica vaginalis in front of the lower third of the hernia. From the great size of the tumor, and from the absence of the usual urgency of the symptoms in strangulated hernia, Mr. Cooper was induced to attempt the division of the stricture without opening the sac; but it adhered too firmly to admit of this being done: it was then laid open, but not to a greater extent than merely to allow of the division of the stricture. From the tympanitic state of the abdomen, all attempts to return the intestine into its cavity were unavailing, and, as it was perfectly free from all adhesions, Mr. Cooper, after a few efforts, determined to leave it within the sac until the bowels had evacuated their contents, and admitted of the return of the hernia. Sutures were then applied, and the wound being dressed in the usual manner, the patient was carried to bed. Fomentations were applied to the whole of the abdomen and scrotum, and a castor-oil enema was administered.

Three o'clock. He complains of great pain in the hernia, and also of the abdomen; pulse frequent, quick and wiry; respiration hurried; no sickness, but constant eructations and tenesmus. From this time until six o'clock, he remained much the same, when the injection returned, unaccompanied by fæces, but tinged with the contents of the colon: this, however, afforded the patient great relief.

At eight o'clock P.M., Mr. Cooper and Mr. Callaway saw him, when his symptoms were, pulse 104, quick and jerking; tongue covered with a slight brown fur; skin warm and dry; abdomen painful on pressure, particularly near the tumor. He was bled to ℥xviij. without fainting, the pulse evidently rising with the abstraction of blood; hirudines xx. were applied to the abdomen; two drachms of Sulphate of Magnesia and gtt. x. Tr. Opium in an ℥iss. of mint water, ordered to be given immediately, and a drachm of Sulphate of Magnesia to be repeated every second hour.

October 27th, eight o'clock A.M. Slept comfortably during the night for at least six hours; he then woke, and took his draught. Has had two fluid motions since last evening. Pulse 120, and sharp; tongue white and dry; much thirst; abdomen more tender; hiccup returned. Two sutures were removed, as the scrotum was tense and inflamed. Twenty-four leeches were applied to the abdomen, and the fomentations to be continued; to take Hyd. Subm. gr. ij., Ext. Opii, gr. i. statim; and continue the sulphate of magnesia.

Twelve o'clock. Pulse not so full; the abdomen less tense, and not so painful; he says he could both hear and feel air passing through the intestine from the hernial sac into the abdomen. Pill and enema repeated.

Four P.M. A healthy, copious motion from the small intestines was produced by the enema: the abdomen is less tense; the pulse more full and soft; skin moist. He felt easy, and said he was sure he should recover.

At five o'clock, Sir A. COOPER, coming accidently to the hospital, was taken by Mr. SCARR, the dresser, to see the patient. He pressed the tumor twice, and each time air was heard to pass from it into the abdomen, which clearly shewed that the intestine was pervious and free from stricture. Sir Astley prescribed the following injection: Ext. Colocynth. comp. ℈ij., Aqua tepid. Oj. statim injic.

Eight P.M. Pulse 126, soft and compressible; tongue moist, and covered with a slight brown fur. The pill of calomel and opium to be repeated.

Eleven P.M. Symptoms still more favorable: abdomen flaccid; tumor soft and yielding; countenance cheerful; pulse 110, no thirst, and feels inclined to sleep. He continued in this state until two o'clock, when violent purging and constant sickness came on to such an extent that, to use his own words, he was on the bedpan all night.

Six o'clock A.M. Purging still continues; countenance anxious; skin bedewed with a cold clammy sweat; pulse so quick and feeble as not to be counted; extremities cold. Forty drops of opium in some brandy were administered.

Seven o'clock. A great quantity of a green fluid was discharged from his mouth and nostrils, and the patient expired forty-two hours after the operation.

*Examination of the body, seven hours after death.* Body yet warm; abdomen distended with gas; scrotum quite livid.

On laying open the abdomen, no marks of inflammation were seen in the general cavity; the intestines were much distended with gas; the parts about the right inguinal ring were adherent to an adjacent portion of the prolapsed gut, which adhesion seemed to limit the inflammation.

The scrotum was examined: the layers down to the sac were softly adhering to one another by means of coagulated blood effused between them, and in the cellular membrane of the lower part of the scrotum there was a very copious effusion of serous lymph. It was found that the tunica vaginalis had been laid open in the operation: it had been dilated so much, in consequence of hydrocele, that it lay at the lower part in front of the sac, which had been divided, therefore, through the tunica vaginalis.

Part of the fluid in the hydrocele escaped during the operation, but there still remained three or four ounces of bloody turbid serum in the cavity; the testicle lay below the sac, and had been exposed in the operation.

On opening the sac and examining the ring, the stricture was found so fully divided that two or three fingers could easily be passed up; the resistance to the return of the gut arising from the distention of the bowels. The sac contained about six inches of small intestine, commencing three inches from the cœcum; there were layers of bloody fibrine in it, and it seemed ecchymosed, and perhaps inflamed; generally speaking, it was adhering to the surrounding sac by soft layers of red lymph, that were easily separated by the finger. There was more of this adhesion at the lower part. The upper extremity of the strictured gut, on being laid open, presented an abrupt elevation, marking the line of the stricture; the lower end, though raised, was less so; the mucous membrane of all the strictured gut was *fully* injected with blood. The mesentery of this gut was also ecchymosed; one considerable patch, which lay at the ring within the abdomen, seemed to mark the adhesion formed between it and the divided ring. The spermatic vessels were found behind the sac in their usual course. The sac itself was inflamed; it was formed by a prolongation of that portion of the peritoneum which lies between the posterior layer of the mesentery and that which ties down the cœcum. There was considerable thickening of the mouth of the sac.

The tunica vaginalis was inflamed; *one spot* was quite gangrenous to an extent of about three quarters of an inch wide, and two or three inches long. The scrotum was infiltrated and commencing to gangrene.

The left inguinal ring was dilated, so as to admit the finger an inch and half; but there was no intestine in this sac.

Liver fatty and pale. Kidneys pale; the other abdominal viscera sound.

*Reported by* Mr. John V. Lewis,
*Dresser, Guy's Hospital.*

